# Factors influencing the bone mineral density in Duroc boars

**DOI:** 10.1186/s40813-023-00318-w

**Published:** 2023-05-23

**Authors:** Lingling Hu, Jinxin Lu, Liangliang Guo, Jiajian Tan, Haiqing Sun, Yuanfei Zhou, Yinghui Wu, Hongkui Wei, Siwen Jiang, Jian Peng

**Affiliations:** 1 Department of Animal Nutrition and Feed Science, College of Animal Science and Technology, Huazhong Agricultural University, Wuhan, 430070 China; 2 YangXiang Joint Stock Company, Guigang, 537000 China; 3 Key Lab of Agricultural Animal Genetics, Breeding and Reproduction of Ministry of Education & Key Lab of Swine Genetics and Breeding of Ministry of Agriculture, Huazhong Agricultural University, Wuhan, 430070 China; 4 The Cooperative Innovation Center for Sustainable Pig Production, Wuhan, 430070 China

**Keywords:** Bone mineral density, Boars, Influencing factors, Leg weakness, Logistic regression

## Abstract

**Background:**

Leg weakness affects animal welfare and is one of the primary reasons for culling of boars. Low bone mineral density (BMD) is one of the primary factors contributing to leg weakness. Low BMD also appeared to be associated with severe bone pain and has the highest risk of skeletal fragility. Surprisingly, few studies have been performed on the factors influencing BMD in pigs. Therefore, the primary aim of this study was to identify the influencing factors on boar BMD. Herein, the BMD data were determined through the use of ultrasonography from 893 Duroc boars. Logistic regression model was utilized in the analysis of BMD, in which the explanatory variables in the model were lines, ages, body weights, backfat thicknesses and serum mineral element concentrations (Ca, P, Mg, Cu, Fe, Zn, Mn, Se, Pb and Cd).

**Results:**

Results showed that factors significantly influencing BMD included serum Ca, P concentrations, ages and backfat thicknesses (*P* < 0.05), in which serum Ca concentrations were positively correlated with BMD (*P* < 0.01), whereas increasing concentrations of serum P decreased BMD (*P* < 0.01). The serum Ca/P ratio showed significant quadratic effects on BMD (r = 0.28, *P* < 0.01), and the Ca/P ratio to achieve the best BMD was determined to be 3.7. Furthermore, BMD also changed with age quadratically (r = 0.40, *P* < 0.01), and reached a peak value around 47 months. Interestingly, a quadratic (r = 0.26, *P* < 0.01) increase in the BMD was observed as backfat thickness increased, and the inflection point was calculated at around 17 mm.

**Conclusion:**

In conclusion, BMD characteristics of boars could be detected by ultrasonic method, and serum Ca, serum P, age, and backfat thickness contributed to the greatest effect on BMD.

**Supplementary Information:**

The online version contains supplementary material available at 10.1186/s40813-023-00318-w

## Background

Leg weakness is a growing concern as it is a common health problem encountered in many pig herds, which affects animal welfare [1, 2], and is one of the main reasons for culling and mortality [3]. Leg weakness prevalence in pig herds ranged from 8.8 to 16.9% [2], and was reported near 12% in boars [4].

Bone mineral density (BMD) is the applicable indices for evaluating bone quality in humans [5], which can be measured by ultrasonic method [3]. Low BMD is associated with severe bone pain. It is among the strongest risk factors for skeletal fragility [6], and is also one of the main causes of leg weakness [3]. Several interrelated factors such as genetics, sex, age, diet, mineral homeostasis and physical activity are thought to be important in human bone [7]. Adipose tissue can influence bone size, BMD, bone mineral content (BMC) and bone microstructure in adolescents [7, 8] and adults [9]. Surprisingly, few studies have been performed on BMD influencing factors in pigs. The majority of studies on porcine bone measurements have focused on BMD, chemical composition as well as the mechanical characteristics [10, 11]. However, the BMD characteristics of boar and the relationship between boar body condition and BMD have not been studied. Though mineral elements, such as Ca and P, influence bone quality and include bone matrix synthesis and mineralization [12], the relationships between boar serum mineral elements and BMD are still poorly understood.

Monitoring the BMD is important to evaluate bone health and prevent osteoporosis in humans [13]. To our knowledge, no direct, large sample studies on the influencing factors of BMD in boars have been reported. Therefore, the aim of this paper was to identify risk factors associated with BMD in boars and to investigate the relationship between influencing factors and BMD to ascertain which factors are the more critical factor in determining BMD.

## Materials and methods

### Animals, housing and diet

A total of 893 Duroc boars (10–67 months) without virus infection were selected for the study and sampled from December 2021 to March 2022. For the purpose of this study, pigs were not distinguishingly raised or treated in any way. And all procedure involving animals were approved by the Animal Management Office of Animal Science and Technology at Huazhong Agricultural University. To obtain the same herd environments, all animals in this study were selected from an artificial insemination centre of Guangxi Yangxiang Co., Ltd., Guangxi Province, China. The experimental boars were housed in individual crates (0.80 × 2.40 × 1.1 m) with concrete flooring that had been slotted. An automated production system, including positive pressure ventilation, automatic feeding and heating systems to control the indoor environment was used (Automated Production Systems, 1004 E. Illinois St. Assumption, IL 62,510, USA). The indoor ambient temperature was maintained at 19.8–25.4 °C, providing additional light when the natural light cycle cannot meet the daylight conditions of 12 h of illumination. Duroc boars were limited to 2.5 kg/d of a common corn-soybean-based commercial feed per day, fed once at 11:00 (Table 1), and accessed to water ad libitum. Veterinarians conducted all diagnoses and related treatments, and each diagnosis and treatment was recorded by farm staff and served as a backup for the analysis performed in this study.

**Table 1:** Composition and calculated nutrient analysis of basal diet (as fed basis)

Item	Diet	Item	Diet
Ingredient, %		Calculated nutrient content	
Corn	42.18	Calculated NE, kcal/kg	2.30
Barley	35.00	Crude protein, %	14.25
Rice bran meal	8.08	Crude fat, %	2.50
Soybean meal, 45%	7.00	Crude ash, %	5.54
Fish meal	4.00	Crude fiber, %	3.46
L-Lysine HCl	0.23	SID^2^ Lys, %	0.75
Methionine	0.09	SID Met, %	0.32
Threonine	0.13	SID Thr, %	0.55
Tryptophan	0.02	SID Trp, %	0.15
Ground limestone	1.11	Total Ca, %	0.82
Monocalcium phosphate	1.07	Total P, %	0.77
Sodium chloride	0.37	Available P, %	0.43
Ammonium propionate	0.08		
Choline	0.14		
Premix^1^	0.44		
Total	100.00		

^1^Premix composition (ad-fed basis): copper,17 mg/Kg; iron, 160 mg/Kg; zinc, 140 mg/Kg; manganese, 50 mg/Kg; iodine, 0.50 mg/Kg; Selenium, 0.50 mg/Kg; and chromium, 0.22 mg/Kg

^2^SID standard ileal digestible; calculated using SID coefficients for the various ingredients obtained from the NRC (2012)

### Backfat thickness

The backfat thickness was determined in triplicate at the last rib (P2: 6.5–7 cm from the last rib's midline [14]) by using ultrasound (Renco LEAN-MEATER, USA) and was operated by the same employee. Boars were split into eight groups on the basis of backfat thickness (≤ 12, 13, 14, 15, 16, 17, 18, and ≥ 19 mm).

### Bone mineral density

An ultrasonic bone densitometer (Sunlight MiniOmni, Israel) was applied to measure the metatarsal BMD in Duroc boar according to a previously published method [3], and the outcome is presented as speed of sound (SOS). To assure the accuracy of the results, system quality check was done before the first measurement of the day. On the metatarsal bone of the boar, the SOS measurements were carried out three to five times, with the average of the readings being utilized as the outcome.

In accordance with the classified recommendation of World Health Organization (WHO) criteria [15], we categorize these boars on the basis of BMD date. Threshold values to categorize individuals as having strong bone, normal bone, osteopenia or osteoporosis were listed in Table 2. Strong bone: SOS > $\overline{\mathrm{X}}$  + SD; normal bone: $\overline{\mathrm{X}}$-SD ≤ SOS ≤ $\overline{\mathrm{X}}$+SD; osteopenia: $\overline{\mathrm{X}}$-2.5 × SD ≤ SOS < $\overline{\mathrm{X}}$-SD; osteoporosis: SOS < $\overline{\mathrm{X}}$-2.5 × SD (Table 2).

**Table 2:** The standard World Health Organization classifications of bone

Classification	Criteria	SOS(m/s)^1^	Symbol
Strong bone	SOS > $\overline{\mathrm{X}}$+SD^2^	> 4442	+ +
Normal bone	$\overline{\mathrm{X}}$-SD ≤ SOS ≤ $\overline{\mathrm{X}}$+SD	3982 ~ 4442	+
Osteopenia	$\overline{\mathrm{X}}$-2.5 × SD ≤ SOS < $\overline{\mathrm{X}}$-SD	3637 ~ 3982	−
Osteoporosis	SOS < $\overline{\mathrm{X}}$-2.5 × SD	< 3637	–

^1^SOS: speed of sound (m/s);

^2^$\overline{\mathrm{X}}$: the mean value of SOS in metatarsal bone of experimental boars; SD: the standard deviation of SOS in metatarsal bone of experimental boars

### Biomarkers of bone metabolism

Blood was drawn and centrifugated for 10 min at 3000 g at room temperature, the resulting supernatant was aspirated and stored at − 80 °C. Biomarkers of bone metabolism in serum were assessed using double antibody sandwich ELISA. The osteocalcin (OCN), C-telopeptide of type I collagen (CTX-I), Type II procollagen carboxy-terminal peptide (PIICP) and C-telopeptide of type II collagen (CTX-II) were assessed using the pig OT/BGP ELISA kit (mlbio, ml002384, China), pig CTX-I ELISA kit (mlbio, ml028171, China), pig PIICP ELISA kit (mlbio, SU-B81025, China) and pig CTX-II ELISA kit (mlbio, SU-B81026, China), respectively, following the manufacturer’s instructions. In brief, the serum samples were thawed on ice. Pipetting was used to gently homogenised them, and diluted (1:5) with sample diluent. 50 μL of diluted sample and standards were added to the plate wells, and set up blank control wells. Except for blank wells, 100 μL of the enzyme-labeling reagent was added to each well. Then, the plates were incubated at 37 °C for 1 h and rinsed five times with the washing buffer. Thereafter, 50 μL of chromogenic solutions A and B were added to wells. The plates were then incubated for 15 min at 37 °C in the dark. Afterwards, each well received 50 μL of stop reagent. A spectrophotometer was used to measure the colour development set at OD 450 nm.

### Serum mineral elements

Inductively coupled plasma mass spectrometry (Agilent 7900, Agilent Technologies, Tokyo) was used to determine the concentrations of Ca, Mg, Cu, Fe, Zn, Mn, Se, Pb and Cd in serum of 893 boars as described previously [16]. Frozen serum samples were thawed at room temperature, then digested with 1:2 high purified HNO_3_ at 80 °C for 4 h until the solution was transparent. After equilibrating to room temperature, the serum samples were diluted 1:50 and filtered to determine Ca, Mg, Cu, Fe, Zn, Mn, Se, Pb, and Cd. During the measurement, scandium, germanium, rhodium, and bismuth with a final concentration of 10 μg/L were added to all samples as internal standards. Isotopes ^44^Ca, ^24^ Mg, ^63^Cu, ^56^Fe, ^64^Zn, ^55^Mn, ^78^Se, ^208^Pb and ^111^Cd were the analytical masses of the normal sensitivity mode of ICP-MS, which employed argon as carrier gas. All solutions were measured three times. To remove any potential traces of metals, all laboratory utensils were treated with 10% HNO_3_ for 24 h and generously cleaned three times with distilled-deionized water.

The concentration of serum inorganic P was measured using commercial kits (Nanjing Jiancheng Bioengineering Institute, C006-1-1, China).

### Statistical analysis

SAS (version 9.2, SAS Inst. Inc., Cary, NC), IBM SPSS (version 26.0, Armonk, NY: IBM Corp.) and GraphPad Prism software (version 8.0, GraphPad Software Inc, San Diego, CA) were applied to conduct all statistical analysis and the difference was considered to reach a significant extent when *P* < 0.05. Results were expressed as mean ± SEM and analysed by One-way ANOVA. The correlations between age, backfat thickness, serum Ca/P ratio and BMD were examined using Pearson correlation matrix.

To identify potential influencing factors for BMD in boars an ordinal logistic regression model was constructed. This model included BMD as a dependent variable as well as the explanatory variables in the model were line, age, body weight, backfat thickness and serum mineral element concentrations (Ca, P, Mg, Cu, Fe, Zn, Mn, Se, Pb and Cd). The highest group was used as the reference category for each variable. First, the variables were checked for multicollinearity, variance inflation factor values over 10 were considered to indicate multicollinearity [17], and those that had a high correlation (correlation coefficient |r|> 0.7) were excluded. Then, all potential variables were fitted into the univariate logistic regression model, and the results from the final model which includes all variates with *P*-values less than 0.1 were reported. Lastly, the potential factors were identified by ordered logistic regression model (*P* < 0.05). The regression coefficients were expressed as odds ratios (OR) with 95% confidence intervals (CI). The formulation of the model is as follows:

$$\begin{aligned} Logit\left( P \right) = & \beta_{0} + \beta_{a} A + \beta_{b} B + \beta_{c} C + \beta_{d} D + \beta_{e} E + \beta_{f} F + \beta_{g} G \\ + \beta_{h} H + \beta_{i} I + \beta_{j} J + \beta_{k} K + \beta_{l} L + \beta_{m} M + \beta_{n} N \\ \end{aligned}$$

where *β*_*0*_ was the intercept. *A*, *B*, *C*, *D*, *E*, *F*, *G*, *H*, *I*, *J*, *K*, *L*, *M* and *N* stood for strain, age, body weight, backfat thickness, serum Ca, serum P, serum Mg, serum Cu, serum Fe, serum Zn, serum Mn, serum Se, serum Pb and serum Cd, respectively. *β*_*a*_ (a containing 2 dummy variables), *β*_*b*_ (b containing 4 dummy variables), *β*_*c*_ (c containing 4 dummy variables), *β*_*d*_ (d containing 8 dummy variables), *β*_*e*_ (e containing 3 dummy variables), β_f_ (f containing 3 dummy variables), *β*_*g*_ (g containing 3 dummy variables), β_h_ (h containing 3 dummy variables), β_i_ (i containing 3 dummy variables), and β_j_ (j containing 3 dummy variables), β_k_ (k containing k dummy variables), β_l_ (l containing 3 dummy variables), β_m_ (m containing 2 dummy variables) and β_n_ (n containing 2 dummy variables) were represented the slope parameters of the included explanatory variables.

## Results

### Bone characteristics of boars

As shown in Table 3, 893 Duroc boars were included in this study, and the BMD ranged from 3542 to 4650 m/s. In accordance with the classified recommendation of WHO [15], the characteristics of bone are shown in Table 3. 9.74% (n = 87) of boars had strong bone, about 71.33% (n = 637) of boars with normal bone, approximately 17.47% of boars had osteopenia (n = 156), and approximately 1.46% of boars were classified as osteoporosis (n = 13) (Table 3).

**Table 3:** The data distribution of BMD in experimental boars

Item	Classification				Total
	Strong bone	Normal bone	Osteopenia	Osteoporosis	
Sample size	87	637	156	13	893
Rate, %	9.74	71.33	17.47	1.46	100

### Identifying the potential factors affecting BMD by Univariate logistic regression

To identify potential influencing factors for BMD in boars a univariate logistic regression model was constructed. The strong bone group was classified as level 1 (n = 87), the normal bone group was level 2 (n = 637), the osteopenia and osteoporosis groups were classified as level 3 (n = 169). We first assessed the correlations between the different variables, including line, age, body weight, backfat thickness, serum Ca, P, Mg, Cu, Fe, Zn, Mn, Se, Pb and Cd. There was no multicollinearity between different explanatory variables (VIF < 10) (See Additional file 1: Table S1). In addition, the correlation coefficients between different explanatory variables were less than 0.7 (See Additional file 1: Fig. S1), thus, all variables were incorporated into the univariate logistic regression analysis model.

As shown in Table 4 and Additional file 1: Table S2, on the basis of the results of univariate logistic regression analysis, the potential influencing factors showing relation with BMD were lines, ages, body weights, backfat thicknesses, serum Ca and P concentrations (*P* < 0.1). Further, these potential risk factors were then considered together in a multiple ordered logistic regression model.

**Table 4:** Analysis of potential factors affecting BMD using univariate logistic regression

Item	1 = Strong	2 = Normal	3 = Osteopenia	*P*-value	OR (95%CI)^1^
*Line*					
0 = Chinese line	47 (54.02%)^3^	550 (86.34%)	142 (84.02%)	< 0.001	2.57 (0.55–1.34)
1 = American line ^ref 2^	40 (45.98%)	87 (13.66%)	27 (15.98%)	–	–
*Age, M*					
1 = ≤ 12	0 (0)	181 (28.41%)	63 (37.28%)	< 0.001	11.65 (1.97–2.94)
2 = 13–24	2 (2.30%)	304 (47.72%)	63 (37.28%)	< 0.001	7.77 (1.60–2.50)
3 = 25–36	13 (14.94%)	64 (10.05%)	15 (8.88%)	< 0.001	3.98 (0.78–1.98)
4 = ≥ 37	72 (82.76%)	88 (13.82%)	28 (16.56%)	–	–
*Body Weight, Kg*					
1 = ≤ 200	0 (0)	125 (19.62%)	41 (24.26%)	< 0.001	6.78 (1.39–2.44)
2 = 201–250	0 (0)	237 (37.21%)	67 (39.64%)	< 0.001	6.07 (1.32–2.29)
3 = 251–300	43 (49.43%)	206 (32.34%)	39 (23.08%)	0.001	2.18 (0.32–1.24)
4 = ≥ 301	44 (50.57%)	69 (10.83%)	22 (13.02%)	–	–
*Backfat thickness, mm*					
1 = ≤ 12	1 (1.15%)	41 (6.44%)	9 (5.33%)	0.762	1.15 (− 0.75–1.02)
2 = 13	1 (1.15%)	140 (21.98%)	45 (26.63%)	0.204	1.58 (− 0.25–1.17)
3 = 14	8 (9.20%)	162 (25.43%)	51 (30.18%)	0.383	1.37 (− 0.39–1.01)
4 = 15	17 (19.54%)	115 (18.05%)	27 (15.98%)	0.505	0.78 (− 0.98–0.48)
5 = 16	27 (31.03%)	85 (13.34%)	11 (6.51%)	0.003	0.31 (− 1.92–0.39)
6 = 17	9 (10.35%)	43 (6.75%)	5 (2.96%)	0.052	0.42 (− 1.75–0.006)
7 = 18	16 (18.39%)	25 (3.93%)	8 (4.73%)	0.003	0.25 (− 2.27–0.48)
8 = ≥ 19	18 (20.69%)	26 (4.08%)	13 (7.69%)	–	–
*Serum Ca, mg/dL*					
1 = ≤ 8	7 (8.05%)	59 (9.26%)	30 (17.75%)	0.072	1.59 (− 0.041–0.97)
2 = 8–11	65 (74.71%)	420 (65.94%)	96 (56.81%)	0.074	0.73 (− 0.65–0.030)
3 = ≥ 11	15 (17.24%)	158 (24.80%)	43 (25.44%)	–	–
*Serum P, mg/dL*					
1 = ≤ 4	42 (48.28%)	125 (19.62%)	26 (15.38%)	< 0.001	0.22 (− 2.10–0.94)
2 = 4–9	45 (51.72%)	459 (72.06%)	122 (72.19%)	0.018	0.55 (− 1.11–0.10)
3 = ≥ 9	0 (0)	53 (8.32%)	21 (12.43%)	–	–

^1^OR = odds ratio, CI = confidence interval

^2^ref = reference

^3^47(54.02%): Outside the parenthesis is the sample size, n = 47, the proportion of the sample size in the group is shown in parentheses, which is 54.02%

### Analysis of BMD-influencing factors using multivariate ordinal logistic regression

As shown in Table 5, the OR and 95% CI for the relationship among lines, ages, body weights, backfat thicknesses, serum Ca and P concentrations, and BMD are presented. The variables that were significant in multivariate logistic regression model on BMD were ages, backfat thicknesses, serum Ca and P concentrations (*P* < 0.05). Boars with age ≤ 12 months (OR: 9.19, 95% CI: 1.39–3.05), 13–24 months (OR: 8.66, 95% CI: 1.50–2.81) and 25–36 months (OR: 4.41, 95% CI: 0.79–2.18) had lower BMD than those with age ≥ 37 months. Boars with backfat thickness ≤ 12 mm (OR: 0.20, 95% CI: − 2.70 to − 0.51), 13 mm (OR: 0.25, 95% CI: − 2.31 to − 0.45), 14 mm (OR: 0.25, 95% CI: − 2.29 to − 0.48), 15 mm (OR: 0.17, 95% CI: − 2.67 to − 0.88), 16 mm (OR: 0.11, 95% CI: − 3.12 to − 1.37), 17 mm (OR: 0.20, 95% CI: − 2.58 to − 0.67) and 18 mm (OR: 0.25, 95% CI: − 2.34 to − 0.46) had higher BMD than those with backfat thickness ≥ 19 mm. In terms of mineral element contents, boars with serum Ca ≤ 8 mg/dL had lower BMD than those with serum Ca ≥ 11 mg/dL (OR: 1.82, 95% CI: 0.07–1.13). Furthermore, boars with serum P ≤ 4 mg/dL had higher BMD than those with serum P ≥ 9 mg/dL (OR: 0.49, 95% CI: − 1.38 to − 0.05).

**Table 5:** Factors affecting BMD were screened by multivariate ordinal logistic regression analysis

Variables	Estimate	SE^1^	OR^2^	95%CI^3^		*P*-value
				Lower	Upper	
Intercept 2	− 2.80	0.50	–	–	–	< 0.001
Intercept 1	1.66	0.49	–	–	–	0.001
*Line*						
0 = Chinese line	0.21	0.22	1.24	− 0.21	0.64	0.32
1 = American line ^ref 4^	0^5^	0	0	0	0	–
*Age*						
1 = ≤ 12	2.22	0.42	9.19	1.39	3.05	< 0.001
2 = 13–24	2.16	0.33	8.66	1.50	2.81	< 0.001
3 = 25–36	1.48	0.35	4.41	0.79	2.18	< 0.001
4 = ≥ 37	0	0	0	0	0	–
*Body weight, Kg*						
1 = ≤ 200	− 0.13	0.50	0.88	− 1.11	0.86	0.80
2 = 201–250	0.07	0.41	1.08	− 0.74	0.88	0.86
3 = 251–300	− 0.24	0.33	0.79	− 0.88	0.41	0.47
4 = ≥ 301	0	0	0	0	0	–
*Backfat, mm*						
1 = ≤ 12	− 1.60	0.56	0.20	− 2.70	− 0.51	0.004
2 = 13	− 1.38	0.48	0.25	− 2.31	− 0.45	0.004
3 = 14	− 1.38	0.46	0.25	− 2.29	− 0.48	0.003
4 = 15	− 1.77	0.46	0.17	− 2.67	− 0.88	< 0.001
5 = 16	− 2.25	0.45	0.11	− 3.12	− 1.37	< 0.001
6 = 17	− 1.62	0.49	0.20	− 2.58	− 0.67	0.001
7 = 18	− 1.40	0.48	0.25	− 2.34	− 0.46	0.004
8 = ≥ 19	0	0	0	0	0	–
*Serum Ca, mg/dL*						
1 = ≤ 8	0.60	0.27	1.82	0.07	1.13	0.03
2 = 8–11	− 0.13	0.18	0.88	− 0.48	0.23	0.48
3 = ≥ 11	0	0	0	0	0	–
*Serum P, mg/dL*						
1 = ≤ 4	− 0.71	0.34	0.49	− 1.38	− 0.05	0.04
2 = 4–9	− 0.26	0.28	0.77	− 0.81	0.29	0.36
3 = ≥ 9	0	0	0	0	0	–

^1^*SE* Standard error

^2^*OR* Odds ratio

^3^*CI* Confidence interval

^4^*ref* reference

^5^“0” represented the reference category for each explanatory variable

### Relationship between serum levels of calcium, phosphate and BMD

**Fig. 1:**
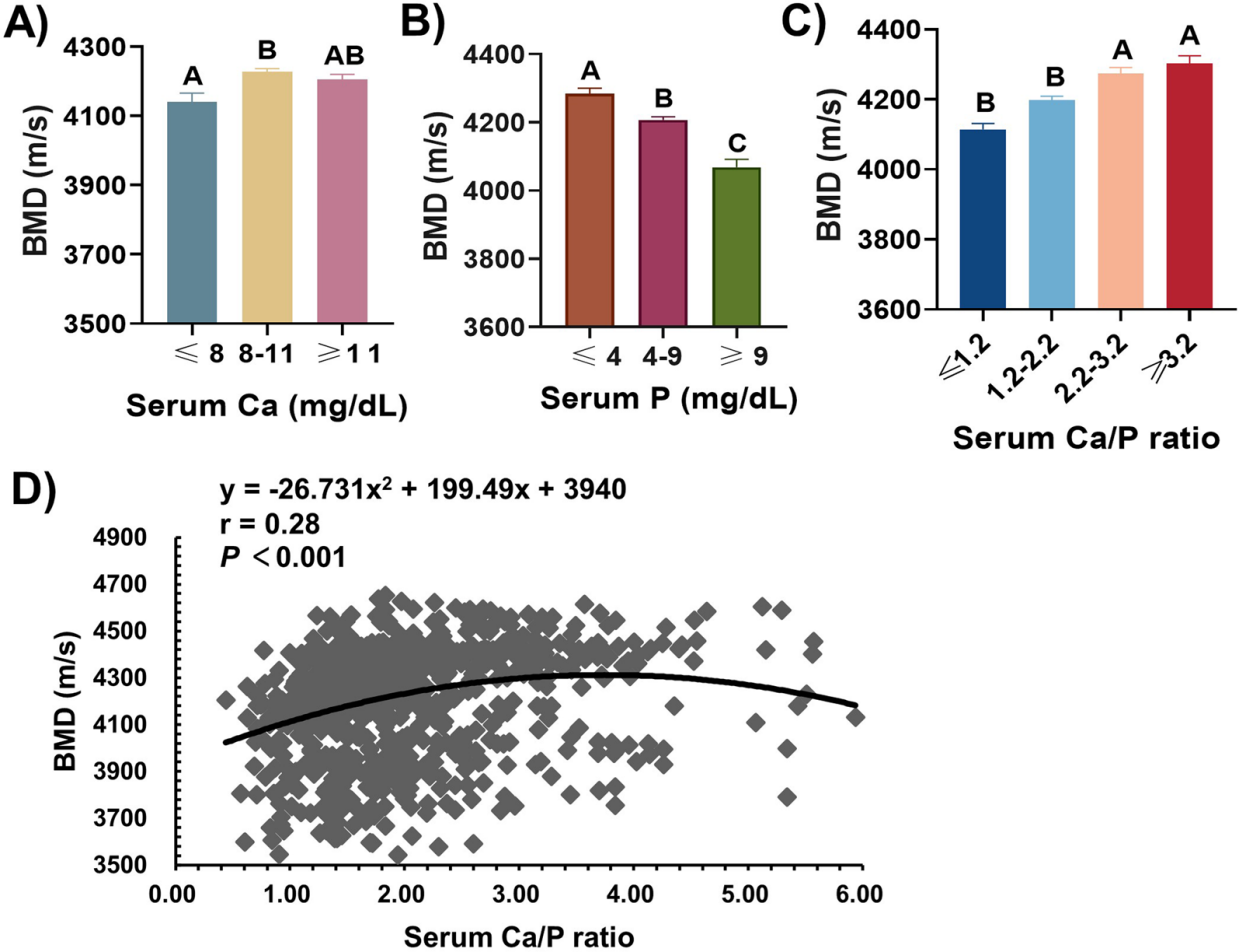
Relationship between serum levels of calcium, phosphate and BMD. **A**–**C** Effects of serum calcium (**A**), phosphate (**B**) levels and serum Ca/P ratio (**C**) on BMD. **D** Quadratic relationship for serum Ca/P ratio and SOS in Duroc boars, each black point represents a sample. Values were expressed as mean ± SEM. **A**, **B** peer data show extremely significant differences without the same letter (*P* < 0.01)

Compared to boars with the lowest serum Ca levels, boars with serum Ca levels above 8 mg/dL had a higher BMD (*P* < 0.01), whereas the BMD was not significantly different in ≥ 11 mg/dL group compared with 8–11 mg/dL group (Fig. 1A). With regard to P, the BMD in the group with the highest serum P concentrations was significantly lower than other groups (*P* < 0.01), the BMD of boars in 4–9 mg/dL group was greater than that of ≥ 9 mg/dL group (Fig. 1B). Additionally, the serum levels of Ca and P were not found to be associated with the levels of serum bone metabolism and cartilage metabolism markers, such as OCN, CTX-I, PIICP, and CTX-II (See Additional file 1: Fig. S2).

Serum Ca and P had inversely related with BMD, and Ca and P homeostasis are closely interrelated. we therefore explored the relationship between serum Ca/P ratio and BMD. As shown in Fig. 1, the serum Ca/P ratio and BMD were positively correlated, increasing BMD was observed for higher Ca/P ratio groups (Fig. 1C). Furthermore, we found a quadratic link between serum Ca/P ratio and BMD (*P* < 0.001), according to the quadratic polynomial regression equation (y = -26.731x^2^ + 199.49x + 3940, r = 0.28, *P* < 0.001), the BMD was the maximum at the serum Ca/P ratio of 3.7 (Fig. 1D). However, serum bone and cartilage metabolism markers were not significantly associated with serum Ca/P ratio measures in any of the five groups (See Additional file 1: Fig. S2).

### Effect of age on bone quality in boars

**Fig. 2:**
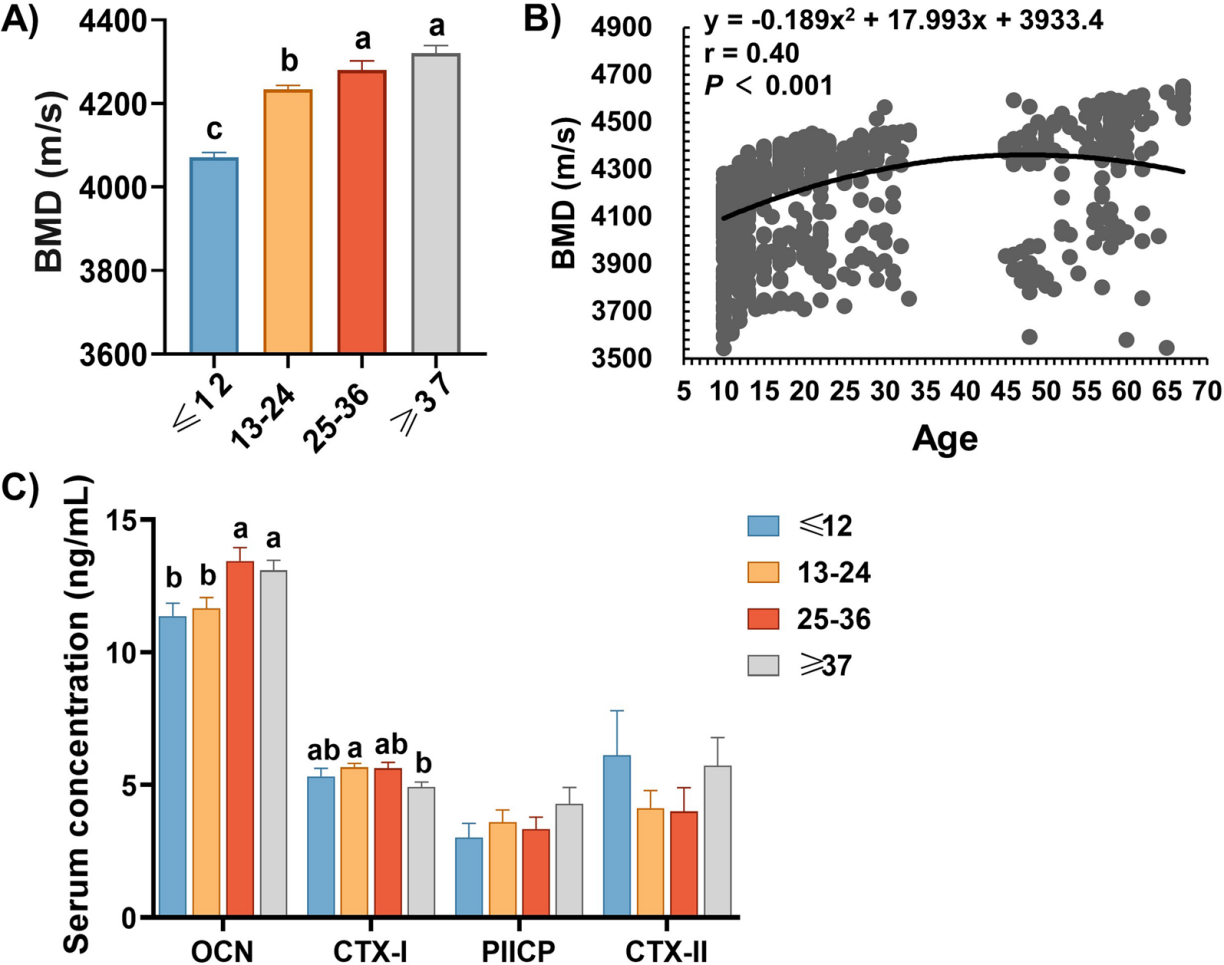
Effect of age on bone quality in boars. **A** Effects of age on BMD. **B** Quadratic relationship for age and BMD in Duroc boars. Each black point represents a sample. **C** Effects of age on serum OCN, CTX-I, PIICP and CTX-II levels. Values were expressed as mean ± SEM. **A**, **B** peer data show significant differences without the same letter (*P* < 0.05)

As shown in Fig. 2A, BMD increased as age increased before 36 months (*P* < 0.01), but BMD did not affect significantly in boars over 37 months. On the scatter plot, the BMD changed with age quadratically (*P* < 0.001). According to the quadratic polynomial regression equation (*y* = − 0.189x^2^ + 17.993x + 3933.4, r = 0.40, *P* < 0.001), the age of 47 months resulted in the maximum BMD (Fig. 2B). Additionally, the BMD and the concentration of osteoblast markers OCN in ≥ 25 months group was higher than that in ≤ 24 months group (Fig. 2B,C), the levels of OCN were not significantly associated between 25–36 months group and ≥ 37 months group (Fig. 2C). While the levels of CTX-I, a marker of bone resorption, were dramatically decreased in ≥ 37 months group compared with that of 13–24 months group (Fig. 2C). The concentrations of cartilage metabolism biomarkers in serum did not differ among each group (Fig. 2C).

### Relationship between backfat thickness and bone quality in boars

**Fig. 3:**
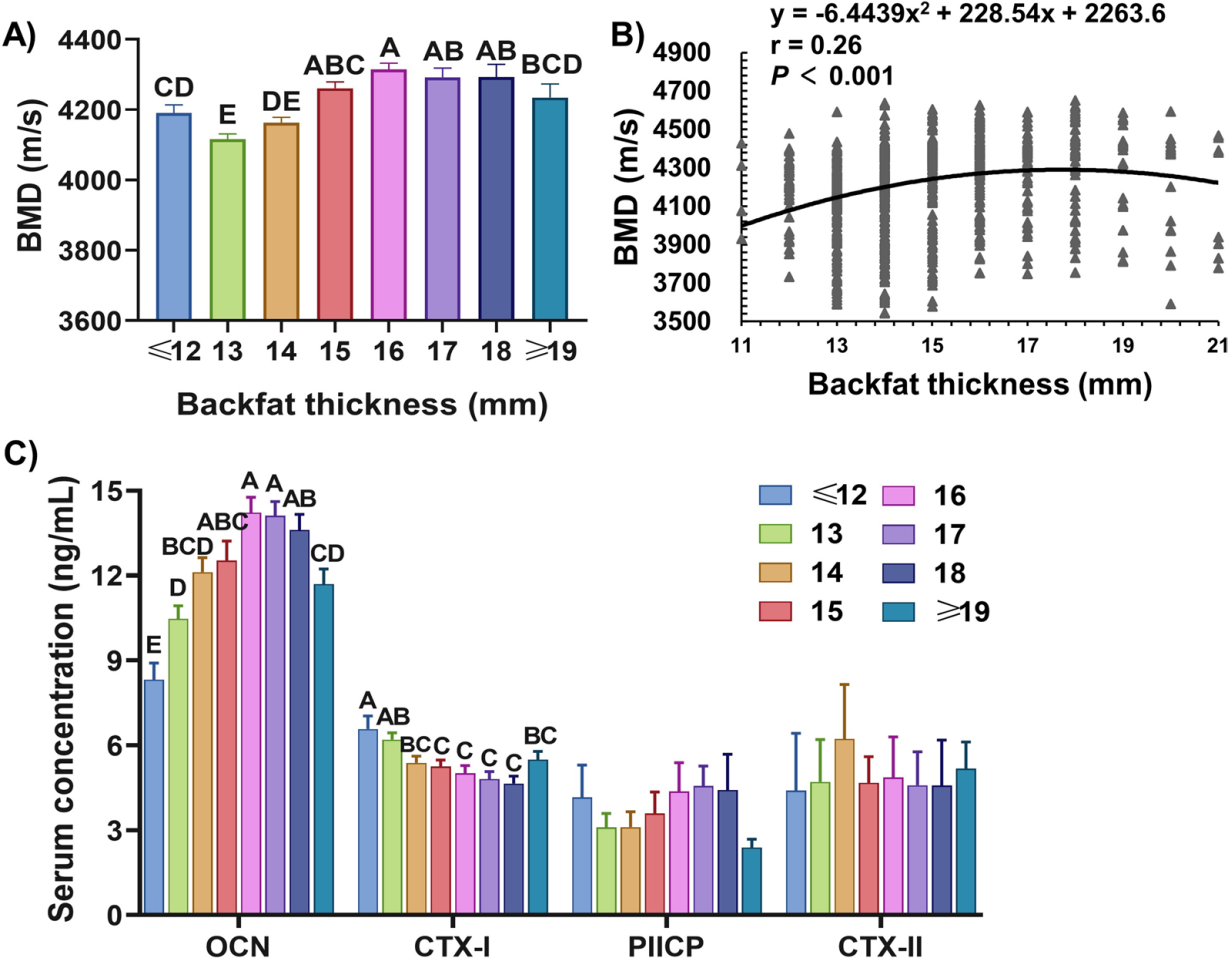
Relationship between backfat thickness and bone quality in boars. **A** Effects of backfat thickness on BMD. **B** Quadratic relationship for backfat thickness and SOS in Duroc boars. Each black point represents a sample. **C** Effects of backfat thickness on serum OCN, CTX-I, PIICP and CTX-II levels. Values were expressed as mean ± SEM. **A**, **B** peer data show extremely significant differences without the same letter (*P* < 0.01)

Figure 3A shows that the difference in BMD was greater in 16 mm,17 mm and 18 mm groups than in ≤ 12 mm, 13 mm and 14 mm groups (*P* < 0.01), and the BMD did not differ among ≤ 12 mm, 14 mm and ≥ 19 mm groups. Further analysis revealed that the BMD increased quadratically as backfat thickness increased (*P* < 0.001). According to the quadratic polynomial regression equation (y = − 6.4439x^2^ + 228.54x + 2263.6, r = 0.26, *P* < 0.001), the maximum BMD was observed at the backfat thickness of 17 mm (Fig. 3B). As shown in Fig. 3C, the concentrations of OCN in serum changes with backfat thickness quadratically, the boars with backfat thickness of less than 13 mm had the lowest OCN concentrations compared to other groups (Fig. 3C). When the backfat thickness was between 16 and 18 mm, the OCN levels showed significantly higher than boars with backfat thickness of over 19 mm and less than 13 mm, respectively (Fig. 3C). And there was no interaction between backfat thickness and cartilage metabolism biomarkers, such as PIICP and CTX-II (Fig. 3C).

## Discussion

Leg weakness poses a serious animal welfare and health problem, which could be caused by low BMD [3], but the influencing factors of BMD in boars remain unclear. The results of the present study showed serum Ca and P levels, age and backfat thickness were the significant factors to influence BMD. However, the changes in line, body weight and serum Mg, Cu, Fe, Zn, Mn, Se, Pb, Cd levels did not have significant effects on BMD. Additionally, the optimal Ca/P ratio for boar BMD is 3.7, and elevation of the ratio from 1.2 to 2.2 could increase BMD. Our results showed a quadratic effect between the BMD and backfat thickness as well as age, boars could obtain the optimum BMD when they were 47 months old and had a backfat thickness of 17 mm.

### The bone characteristic of boars

In the current investigation, the BMD in boars ranged from 3542 to 4650 m/s, which was consistent with previous literature [3, 18], confirming the reliability of our data. But the SOS of BMD from our datasets was higher than humans [19, 20]. This finding is similar to Inui [21] who proposed the BMD of minipigs as recognized by dual-energy X-ray (DXA) had higher bone mass and denser trabecular network than humans. The species differences may arise from multiple metabolic and hormonal changes [21]. And comparing the results of these studies, it is important to consider differences in methods, such as using DXA, computed tomography and ultrasonic methods. We also found approximately one in five (18.93%) of boars with low BMD, this result suggested that controlling risk factors identified in our study may have great value in improving boar bone quality.

### Relationship between serum levels of calcium, phosphate and BMD

In agreement with previous studies [7], the data presented here confirm that BMD is associated with a number of variables, including serum Ca, P levels, age and backfat thickness. But line, body weight and serum Mg, Cu, Fe, Zn, Mn, Se, Pb, Cd levels had no effect on boar BMD. This was in part because other significant influencing factors were more explanatory of BMD than line, body weight and serum Mg, Cu, Fe, Zn, Mn, Se, Pb, Cd levels.

Literature suggests there are established relationship between Ca, P and bone development and mineralization [22]. The results of the logistic regression model show that boars with the lowest serum Ca concentrations were more likely to exhibit low BMD. This conclusion is consistent with the view of others, who discovered that blood Ca values decline in osteoporotic patients and ovariectomized rats [23, 24]. With regard to P, high levels of P would have an adverse effect on bone health. Recently, it discovered serum P was associated with elevated fracture risk, with each 1 mg/dL rise in serum P translating into a 47% higher risk of fracture [25]. Low Ca and high P levels have an adverse effect on bone possibly through increased parathyroid hormone and osteopontin levels, which lead to increased bone resorption and decreased osteogenesis [12, 26].

In addition, we found that the relationship between Ca/P ratio and BMD is linear up to 3.7, after which any increase in Ca/P ratio did not result in an increase in BMD. Actually, in clinical research, it has been proposed that the Ca/P ratio may be a valuable index as a predictive and prognostic factor for a variety of pathological and pre-pathological disorders [27, 28]. Therefore, more mechanistic research is merited.

### Effect of age on bone quality in boars

Age explained much of the variation in bone development, as previously reported at several anatomical locations in humans [29], but very limited data on the age-related changes in bone quality of boars. Physiologically, the closure of the growth plates takes place at approximately 18 months in pigs [30]. In humans, growth plate closure is used to differentiate between adolescence and adulthood, as growth plates in the scapula fuse last, at about 20 years of age on average [31]. Thus, during the adult phase, one pig month is equivalent to one human year. In our study, the BMD was gradually increased before 47 months, and 47-month-old boars are similar to humans in middle age, respectively. Similarly, in both women and men, it was previously found that BMD rise before middle age, and then steadily decreased after middle age [5, 32]. Therefore, the aforementioned data suggests that prevention of bone loss reduction of fracture risk are particularly important for boars over 47 months of age.

### Relationship between backfat thickness and bone quality in boars

Maintaining an optimal body condition of boars will not only improve animal welfare but will also maximize reproductive efficiency and longevity [14]. Robert Charette [33] reported that backfat thickness level was a more objective and accurate parameter to evaluate the body condition of pigs. Our result showed a quadratic effect between the backfat thickness and BMD, the highest BMD was obtained when backfat thickness was around 17 mm. Qiao et al. [9] observed that obese people had a higher lumbar BMD. When absolute value is taken into account, Wetzsteon et al. [34] found that bone strength is higher in obese children. On the other hand, Farr et al. [8] reported that obese adolescents had reduced BMD and bone strength, which could provide insight into why obese adolescents suffer from more fractures than normal ones. Mosca et al. [35] reported a negative relationship between body fat and BMD in males. Moreover, the growing-finishing pigs with a higher body fat percentage at 56-115d had lower BMC and BMD [10]. In our study, boars with backfat thicknesses between 16 and 18 mm had higher BMD than boars with backfat thicknesses of over 19 mm and less than 13 mm. On a physiological level, body fat content supports bone health, while a high one has the opposite impact [10]. Therefore, boar with backfat thickness should be kept within a desirable range to ensure the best bone quality.

Although our study did not find a direct relationship between backfat thickness and BMD, some literature pointed out a physiologically predominant role of the adipose tissue in the body energy balance by releasing leptin, adiponectin, resistin as well as proinflammatory molecules [36, 37]. Moreover, all of these cytokines can directly or indirectly influence bone development demonstrating the dynamic nature of the bone to adipose tissue [38]. Taken together, in-depth studies are warranted to clarify the mechanisms of adipose tissue on bone mineralization, bone microstructure, and bone strength [10].

## Conclusions

In conclusion, our results provide a better understanding of the factors affecting BMD in boars. These risk factors were serum Ca, P levels, age and backfat thickness. Increasing the serum Ca/P ratio, age or backfat thickness have quadratic effect on BMD. Serum Ca/P ratio of 3.7, the age of 47 months and the backfat thickness of 17 mm contributed to the highest value of BMD. Therefore, it is an important approach to optimize bone quality to regulate the appropriate serum Ca/P ratio, determine the appropriate age and adjust the appropriate backfat thickness through nutritional means and management strategies.

## Supplementary Information

**Additional file 1**. **Table 1.** Multicollinearity analysis table of variables in this study. **Table 2.** Analysis of potential factors affecting BMD using univariate logistic regression. **Figure 1.** Heat map from the correlations between the different univariates. **Figure 2.** Association between serum levels of calcium, phosphate and BMD

## Data Availability

The datasets used and/or analyzed during the current study are available from the corresponding author on reasonable request.
