# Supplementary material for: Factors influencing the bone mineral density in Duroc boars
Source: Porcine Health Manag. 2023 May 23;9:22. doi: 10.1186/s40813-023-00318-w (PMC10207601; doi:10.1186/s40813-023-00318-w)
Supplement: Supplementary file 1 — Additional file 1. Table 1. Multicollinearity analysis table of variables in this study. Table 2. Analysis of potential factors affecting BMD using univariate logistic regression. Figure 1. Heat map from the correlations between the different univariates. Figure 2. Association between serum levels of calcium, phosphate and BMD [file 40813_2023_318_MOESM1_ESM.docx]

**Supplementary Table 1.** Multicollinearity analysis table of variables in this study (n=893)

| Item | Tolerance | VIF^1^ |
| --- | --- | --- |
| Line | 0.85 | 1.18 |
| Age | 0.29 | 3.40 |
| Body Weight | 0.24 | 4.10 |
| Backfat thickness | 0.46 | 2.17 |
| Serum Ca | 0.61 | 1.64 |
| Serum P | 0.70 | 1.42 |
| Serum Mg | 0.56 | 1.78 |
| Serum Cu | 0.85 | 1.18 |
| Serum Fe | 0.90 | 1.11 |
| Serum Zn | 0.94 | 1.06 |
| Serum Mn | 0.85 | 1.18 |
| Serum Se | 0.90 | 1.11 |
| Serum Pb | 0.97 | 1.03 |
| Serum Cd | 0.83 | 1.20 |

^1^VIF= Variance inflation factor

**Supplementary Table 2.** Analysis of potential factors affecting BMD using univariate logistic regression (n=893)

| Item | 1=Strong | 2=Normal | 3=Osteopenia | | *P-*value | OR (95%CI)^1^ | |
| --- | --- | --- | --- | --- | --- | --- | --- |
| **Serum Mg, mg/L** | |  | |  |  |  |  |
| 1=≤18 | 17(19.54%)^3^ | 160(25.12%) | 41(24.26%) | | 0.408 | 1.22(-0.27-0.67) | |
| 2=18-22 | 56(64.37%) | 381(59.81%) | 106(62.72%) | | 0.527 | 1.14(-0.28-0.55) | |
| 3=≥22 ^ref 2^ | 14(16.09%) | 96(15.07%) | 22(13.02%) | | - | - | |
| **Serum Cu, mg/L** | |  | |  |  |  |  |
| 1=≤2.0 | 41(47.12%) | 278(43.64%) | 69(40.83%) | | 0.358 | 0.81(-0.67-0.24) | |
| 2=2.0-2.5 | 38(43.68%) | 278(43.64%) | 78(46.15%) | | - | 0.91(-0.55-0.36) | |
| 3=≥2.5 | 8(9.20%) | 81(12.72%) | 22(13.02%) | | - | - | |
| **Serum Fe, mg/L** | |  | |  |  |  |  |
| 1=≤1.0 | 44(50.57%) | 299(46.94%) | 89(52.66%) | | 0.711 | 0.91(-0.58-0.40) | |
| 2=1.0-2.5 | 37(42.53%) | 270(42.39%) | 62(36.69%) | | 0.323 | 0.78(-0.75-0.25) | |
| 3=≥2.5 | 6(6.90%) | 68(10.67%) | 18(10.65%) | | - | - | |
| **Serum Zn, mg/L** | |  | |  |  |  |  |
| 1=≤0.5 | 33(37.93%) | 249(39.09%) | 79(46.75%) | | 0.245 | 1.21(-0.13-0.52) | |
| 2=0.5-1.0 | 21(24.14%) | 142(22.29%) | 29(17.16%) | | 0.390 | 0.84(-0.56-0.22) | |
| 3=≥1.0 | 33(37.93%) | 246(38.62%) | 61(36.09%) | | - | - | |
| **Serum Mn, μg/L** | |  | |  |  |  |  |
| 1=0 | 41(47.13%) | 407(63.89%) | 103(60.95%) | | 0.578 | 1.12(-0.28-0.50) | |
| 2=0-30 | 22(25.29%) | 137(21.51%) | 30(17.75%) | | 0.467 | 0.84(-0.64-0.30) | |
| 3=≥30 | 24(27.58%) | 93(14.60%) | 36(21.30%) | | - | - | |
| **Serum Se, μg/L** | |  | |  |  |  |  |
| 1=≤150 | 7(8.05%) | 161(25.27%) | 43(25.44%) | | 0.407 | 1.21(-0.27-0.65) | |
| 2=150-300 | 64(73.56%) | 382(59.97%) | 94(55.62%) | | 0.225 | 0.78(-0.65-0.15) | |
| 3=≥300 | 16(18.39%) | 94(14.76%) | 32(18.93%) | | - | - | |
| **Serum Pb, μg/L** | |  | |  |  |  |  |
| 1=0 | 65(74.71%) | 482(75.67%) | 132(78.11%) | | 0.485 | 1.13(-0.22-0.46) | |
| 2=＞0 | 22(25.29%) | 155(24.33%) | 37(21.89%) | | - | - | |
| **Serum Cd, μg/L** | |  | |  |  |  |  |
| 1=0 | 71(81.61%) | 576(90.42%) | 145(85.80%) | | 0.886 | 1.03(-0.42-0.49) | |
| 2=＞0 | 16(18.39%) | 61(9.58%) | 24(14.20%) | | - | - | |

^1^OR=odds ratio, CI=confidence interval.

^2^Ref=reference

^3^17(19.54%): Outside the parenthesis is the sample size, n=17, the proportion of the sample size in the group is shown in parentheses, which is 19.54%.


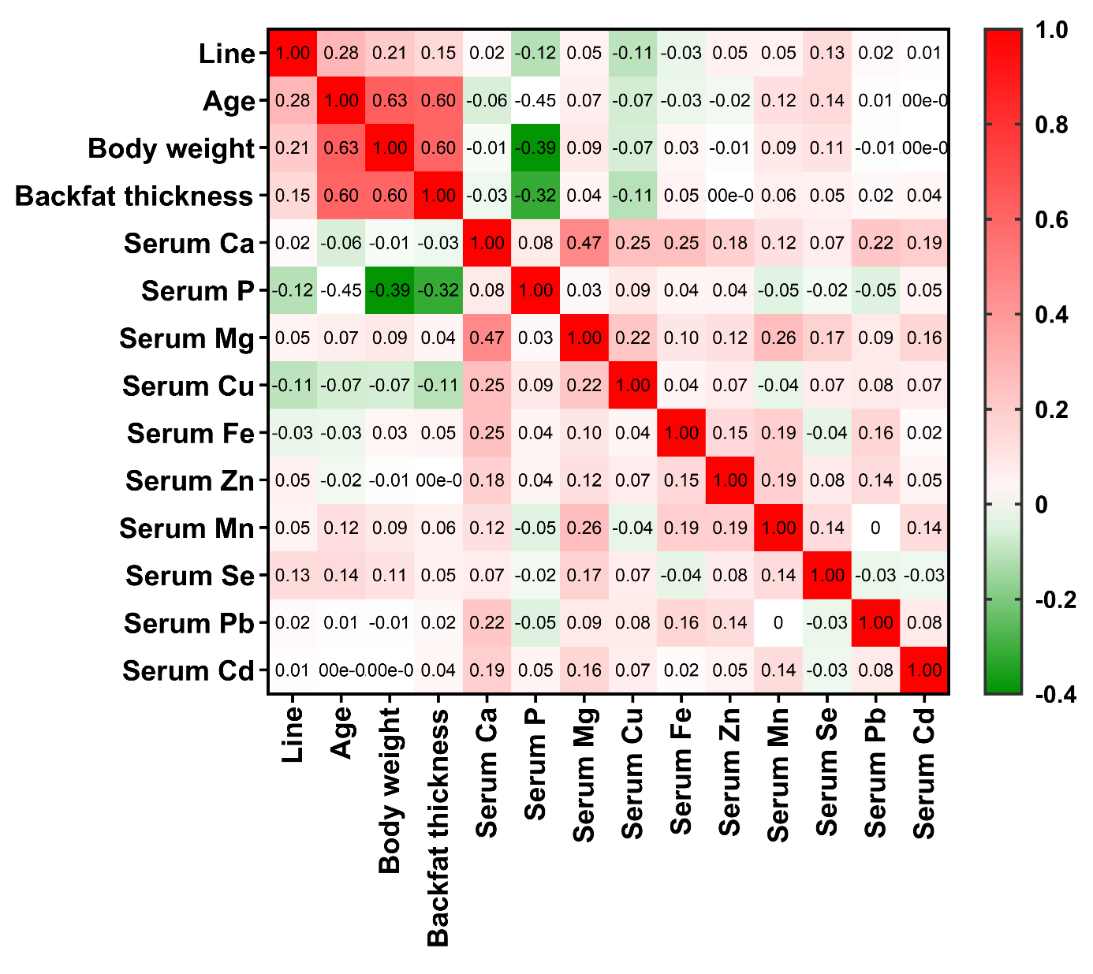


**Supplementary Figure 1.** Heat map from the correlations between the different univariates (line, age, body weight, backfat thickness and serum concentrations of Ca, P, Mg, Cu, Fe, Zn, Mn, Se, Pb, Cd) of Duroc boars (n=893).

**
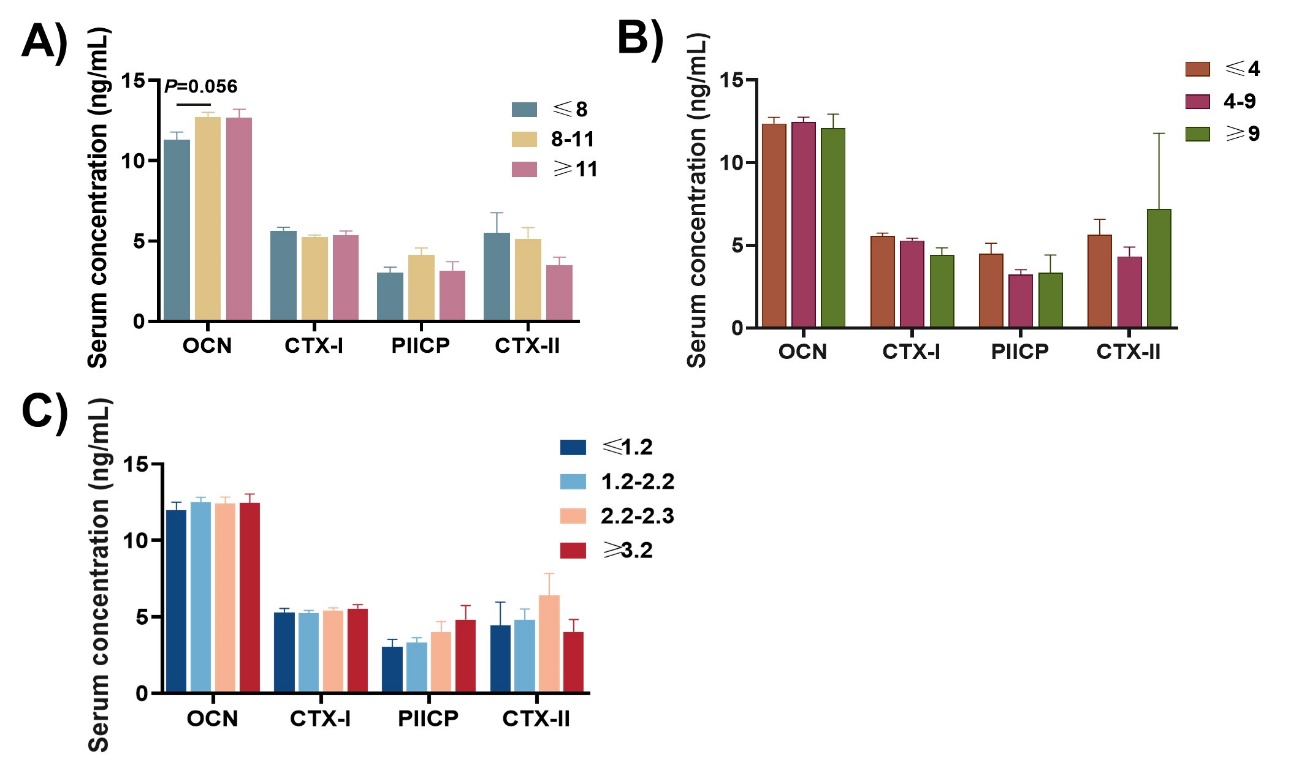
**

**Supplementary Figure 2.** Association between serum levels of calcium, phosphate and BMD. (A-C) Effects of serum calcium (A), phosphate (B) levels and serum Ca/P ratio (C) on serum OCN, CTX-I, PIICP and CTX-II levels. Values were expressed as mean ± SEM.
